# Neural activities during the Processing of unattended and unseen emotional faces: a voxel-wise Meta-analysis

**DOI:** 10.1007/s11682-022-00697-8

**Published:** 2022-06-23

**Authors:** Zeguo Qiu, Xue Lei, Stefanie I. Becker, Alan J. Pegna

**Affiliations:** School of Psychology, The University of Queensland, Brisbane, 4072 Australia

**Keywords:** Visual awareness, Unconscious processing, Emotional faces, fMRI, meta-analysis

## Abstract

Voxel-wise meta-analyses of task-evoked regional activity were conducted for healthy individuals during the unconscious processing of emotional and neutral faces with an aim to examine whether and how different experimental paradigms influenced brain activation patterns. Studies were categorized into sensory and attentional unawareness paradigms. Thirty-four fMRI studies including 883 healthy participants were identified. Across experimental paradigms, unaware emotional faces elicited stronger activation of the limbic system, striatum, inferior frontal gyrus, insula and the temporal lobe, compared to unaware neutral faces. Crucially, in attentional unawareness paradigms, unattended emotional faces elicited a right-lateralized increased activation (i.e., right amygdala, right temporal pole), suggesting a right hemisphere dominance for processing emotional faces during inattention. By contrast, in sensory unawareness paradigms, unseen emotional faces elicited increased activation of the left striatum, the left amygdala and the right middle temporal gyrus. Additionally, across paradigms, unconsciously processed positive emotions were found associated with more activation in temporal and parietal cortices whereas unconsciously processed negative emotions elicited stronger activation in subcortical regions, compared to neutral faces.

**Supplementary Information:**

The online version contains supplementary material available at 10.1007/s11682-022-00697-8.

## Introduction

Emotional human faces are important in our daily life. Negative emotions like fearful and angry expressions tend to indicate potential threat in our surroundings (Fox et al., 2000; Phelps et al., 2006), and positive emotions like happy faces play a crucial role in social interactions (Beaudry et al., 2014; Wirth & Wentura, 2020). Moreover, a processing bias is observed towards emotional faces, even when the faces are presented below our awareness threshold (e.g., Del Zotto & Pegna, 2015; Pegna et al., 2011).

Brain imaging techniques, especially functional magnetic resonance imaging (fMRI), have been widely used to reveal the neural networks involved in unconscious processing of emotional faces. Emotional faces processed implicitly, as opposed to explicitly, have been found to activate the amygdala, the insula, and fronto-occipital areas more strongly than neutral faces (for a review see Tao et al., 2021). Activations of these regions have been suggested to be important for the neural representation of (LeDoux, 2000), and emotional reactivity to (Gruber et al., 2016), emotional information without visual awareness. In particular, negative emotions such as fear can be conveyed through a subcortical pathway that runs in parallel to the cortical, geniculo-striate route (Compton, 2003; LeDoux, 2000; Tamietto & De Gelder, 2010). The amygdala, a target region for this subcortical pathway, has been found to be sensitive to the emotional expression of faces, even when the faces are not consciously processed (Diano et al., 2017; Whalen et al., 1998). Convergent evidence was obtained from studies on patients with brain lesions. Indeed, patients suffering from cortical blindness following lesions of their primary visual cortex have been reported to guess emotional expressions of faces at an above-chance level, a phenomenon termed affective blindsight (De Gelder et al., 1999). Importantly, in affective blindsight, emotional faces were found to activate the right amygdala (Burra et al., 2017; Pegna et al., 2005). The absence of the primary visual cortex suggested that threat-related signals like fear must reach the amygdala through a subcortical pathway (i.e., the colliculus-pulvinar-subcortical pathway; LeDoux, 2000; Méndez-Bértolo et al., 2016).

In heathy individuals, unconscious processing of emotional faces is mainly examined with two broad types of experimental paradigms. In *sensory unawareness* paradigms, stimuli are rendered invisible to the participants either by backward masking or interocular suppression. In *attentional unawareness* paradigms, stimuli are rendered irrelevant to participants’ experimental task and hence are unattended (e.g., Diano et al., 2017). While attentional unawareness encompasses active attentional suppression over the stimuli presented, sensory unawareness is achieved by making stimuli undetectable at the perceptual level. Findings on the underlying neural mechanisms of these paradigms are inconclusive with mixed results reported across different paradigms and sometimes within a same category of paradigms.

In sensory unawareness paradigms, some researchers found increased activation of the amygdala and a wide range of cortical regions for unseen emotional faces, compared to unseen neutral faces (e.g., Chen et al., 2017; Dannlowski et al., 2007). However, others found that unseen emotional faces elicited stronger activity only in cortical regions (e.g., anterior cingulate cortex; Duval et al., 2013). Several studies even found no differences in activation between unseen emotional and neutral faces (e.g., Amting et al., 2010; Chen et al., 2015).

Similar to certain sensory unawareness findings, attentional unawareness paradigms found stronger activations in frontal and temporal areas (e.g., Holtmann et al., 2013; Vuilleumier et al., 2001) and the amygdala (e.g., Schulte Holthausen et al., 2016; Pichon et al., 2012) for unattended emotional compared to neutral faces. Additional subcortical regions including the thalamus and the striatum showed stronger activity to unattended emotional compared to neutral faces (e.g., Holtmann et al., 2013). Moreover, reduced activity in V5 and occipital regions were reported for unattended emotional compared to neutral faces (Attar et al., 2010; Holtmann et al., 2013).

Considering the similarities and noticeable differences between the findings from different experimental paradigms, a systematic examination is needed to compare brain activation patterns between unseen and unattended emotional faces. In a previous meta-analysis, Shi et al. (2013) compared the activation patterns between two types of paradigms for implicitly processed faces. They found that inattention tasks were associated with increased activation of more cortical regions (e.g., fusiform gyrus, inferior frontal gyrus and precuneus) than masking experiments when emotional faces were compared to neutral ones (Shi et al., 2013). The activation of cortical regions by inattention tasks, especially regions in the dorsal attention network (i.e., precuneus), was interpreted as evidence that inattention tasks are associated with a later stage of face processing, which activates the dorsal attention network (Shi et al., 2013).

While these findings are important, this previous meta-analysis did not provide clear and direct examinations on unconscious processing of emotion. Instead, unconscious processing was treated in the same manner as implicit processing (Shi et al., 2013). For example, studies where participants were instructed to judge the gender or age of the faces were considered as a type of implicit processing of the emotion and were included in the analysis. However, given that the faces were still task-relevant and had to be attended, it is unclear to what extent emotion was indeed unattended and suppressed from awareness. Therefore, while previous results using inattention paradigms revealed the neural mechanisms underlying the processing of *task-irrelevant emotions*, it is not known whether these findings would apply if the faces themselves were unattended (i.e., *task-irrelevant faces*). A more focused investigation of unconscious processing should therefore examine unattended faces, rather than unattended emotions.

In addition to the effects caused by dissimilarities in experimental settings, different neural networks may be involved in processing emotions of different valences. Some behavioral research has found that happy faces can be recognized more accurately than other emotions including fear, anger, sadness and disgust when presented very briefly (e.g., 25 ms; Calvo & Lundqvist, 2008). However, an EEG study found that, when compared to happy faces, fearful faces enhanced the amplitude of an early electrophysiological marker related to visual processing (i.e., C1; Zhu & Luo, 2012). Using neuroimaging to directly compare the regions activated by unconsciously processed happy and sad faces, Juruena et al. (2010) found that the amygdala and hippocampus were more strongly activated by masked happy faces compared to masked sad faces. Other neuroimaging studies found similar results when comparing unaware positive or negative emotions against a neutral face (e.g., Faivre et al., 2012; Suslow et al., 2019). Therefore, while both positive and negative emotions can be processed without awareness, there are some inconsistencies in whether the two categories are similarly prioritized during visual processing. Specifically, it remains unknown how the unconscious processing of positive and negative emotions differ regarding their underlying neural networks.

The current meta-analysis aimed to examine systematically the previous findings on unconscious processing of emotional faces by first comparing brain activation patterns between unaware emotional and neutral faces across all experimental paradigms. We then compared brain activation patterns associated with sensory unawareness (i.e., masking and binocular rivalry) and attentional unawareness paradigms (i.e., inattention tasks). Lastly, we examined whether positive emotions (e.g., happy faces) and negative emotions (e.g., fearful, angry, sad and disgusted faces) were associated with different brain activation patterns without visual awareness.

## Method

### Study selection

We searched Scopus, PubMed and Web of Science for articles published in English before August 16th, 2021, using the following terms and their derivatives: “fMRI”; AND “masking” OR “inattention” OR “dual task” OR “binocular rivalry” OR “continuous flash suppression” OR “unconscious” OR “subliminal” OR “priming OR “attentional blink”; AND “emotional” OR “threatening” OR “faces”. The reference lists of relevant review and meta-analysis articles were also examined to include additional papers.

A study was included if it: (1) was published in English, in a peer-reviewed journal; (2) used fMRI; (3) included healthy human participants; (4) compared neural activation between emotional faces and neutral faces in conditions where participants were not aware of the stimuli; (5) conducted whole-brain analyses in the form of three-dimensional coordinates in standard stereotactic coordinate space (i.e., Montreal Neurological Institute or Talairach).

A study was excluded if it: (1) used the same data as other included studies; (2) investigated connectivity or used diffusion tensor imaging; (3) was a resting-state fMRI study.

In the current meta-analysis, we only included studies where the faces were unattended (i.e., excluding situations where non-emotional aspects of the faces, such as gender, were still attended). Also, we only included studies that contrasted emotional faces against neutral faces to obtain emotion-specific results.

**Fig. 1:**
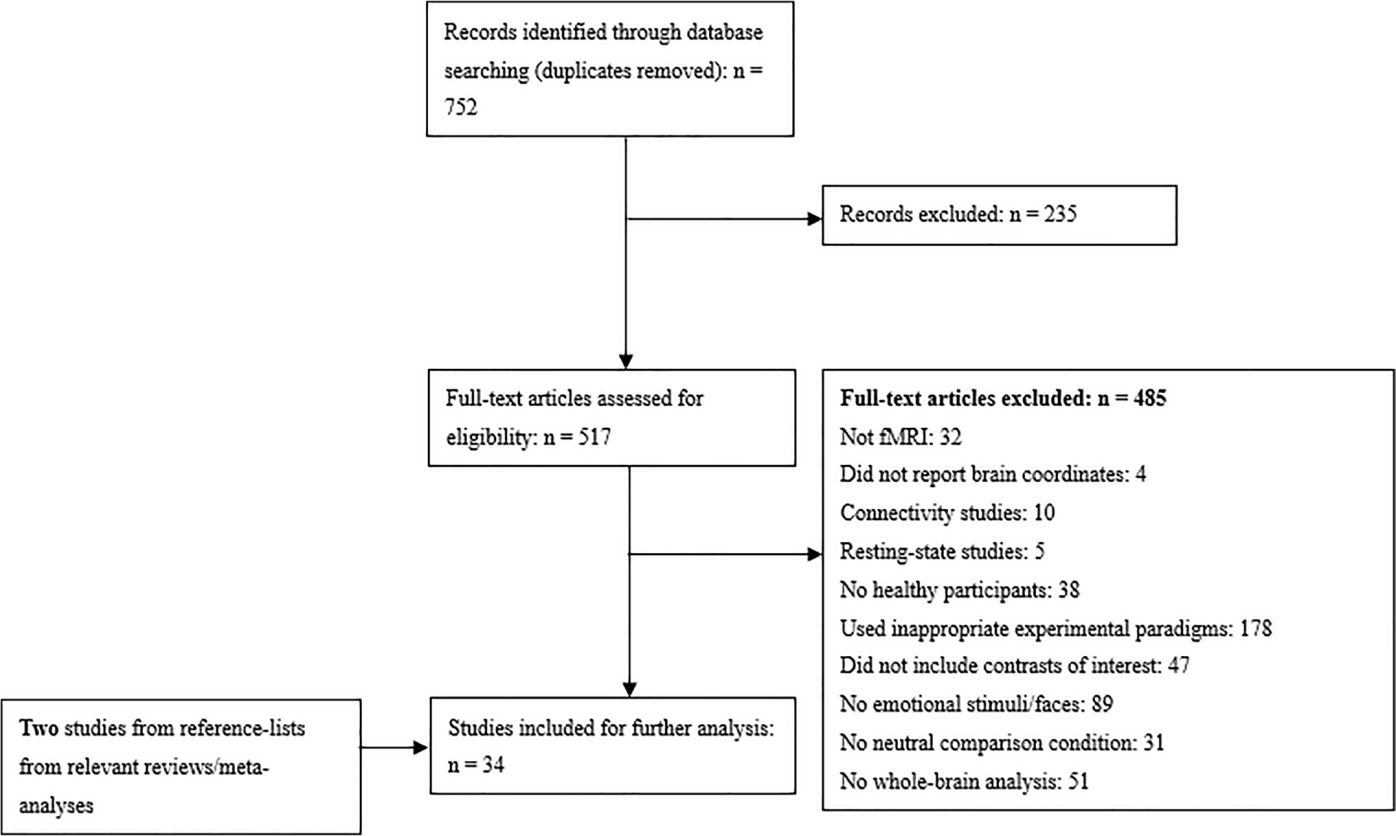
PRISMA Flow Diagram of Study Selection

Quality assessment of each study included was conducted with a 7-point checklist (supplementary Table S1). Information including image acquisition techniques were presented in the Table S2. The current study was performed according to the Meta-analysis of Observational Studies in Epidemiology guidelines (Stroup et al., 2000). See Fig. 1 for the PRISMA Flow Diagram on the study selection for this meta-analysis.

### Data analysis

#### Voxel-wise meta-analysis

We used the Seed-based d Mapping with Permutation of Subject Images (SDM-PSI) software package (version 6.21; http://www.sdmproject.com/software) to perform meta-analyses on the different neural activation patterns for unaware emotional faces and unaware neutral faces. The SDM-PSI method allows the combination of statistical parametric maps and peak coordinates originally reported in individual studies (Albajes-Eizagirre et al., 2019). By using multiple imputation and the threshold-free cluster enhancement (TFCE) statistics, the SDM-PSI provides a less biased estimation of the population effect size (Albajes-Eizagirre et al., 2019; Bossier et al., 2018).

Briefly, we first extracted peak coordinates and effect sizes (e.g., *t* values, *z* values) of the different hemodynamic activity between unaware emotional and unaware neutral faces from each individual study. *Z* scores reported as effect sizes were converted to *t*-values using an online converter (http://www.sdmproject.com/utilities/?show=Statistics). Second, the maps of lower and upper bounds of the effect sizes for all voxels were estimated using an anisotropic Gaussian kernel, which improves the plausibility of the maps by allocating different values to distinct voxels of a peak dependent on relevant spatial correlations. To control for false-positive results, the default kernel size (full anisotropy = 1) and thresholds were used (full width at half maximum [FWHM] = 20 mm and voxel = 2 mm; see Albajes-Eizagirre et al., 2019; Radua et al., 2015). Third, the most likely effect size and the corresponding standard error were estimated using multiple imputations (50 imputations) of a random-effects general linear model to create the mean map (Bossier et al., 2018; Radua et al., 2012). Finally, common permutation tests (1000 permutations) were used to perform family-wise error correction for multiple comparisons in combination with a TFCE in statistical thresholding (*p* < .05 and cluster extent = 10 voxels). As a result, the included studies were weighted differentially based on their sample sizes, between-study heterogeneities and intra-study variances, increasing the contributions of the studies with smaller variance or larger sample size (Radua & Mataix-Cols, 2012).

We contrasted emotional faces, either rendered unseen (by masking or binocular rivalry) or unattended (by inattention), to unseen or unattended neutral faces.

Twenty-four out of 34 studies described their measurements on participants’ awareness of the stimuli in the papers and analyzed data only from participants who reported no awareness of the stimuli. We ran an additional analysis using data from this subgroup across experimental paradigms. Because the results from this additional analysis (Table S3) remained largely the same as the analysis using all available datasets, below we report the results from all 34 datasets to provide a more comprehensive examinations of the effects of interest.

#### Subgroup analyses

To investigate the activation patterns associated with different experimental paradigms, we separated the datasets into unseen (masking or binocular rivalry) and unattended (inattention) subgroups and conducted meta-analytic comparisons between unaware emotional and neutral faces separately for the two groups.

To investigate the activation patterns associated with different emotions, we separated the datasets into positive emotion and negative emotion groups and conducted meta-analytic positive-neutral and negative-neutral comparisons separately.

#### Jackknife sensitivity analysis

We assessed the replicability of the results by conducting a systematic whole-brain voxel-based Jackknife sensitivity analysis. Specifically, we repeated the main statistical analysis while removing one study each time (Radua & Mataix-Cols, 2009).

#### Analyses of heterogeneity and publication bias

We performed the *I*^*2*^ statistics heterogeneity analysis to investigate unexplained between-study variability with *I*^*2*^ < 50% indicating low heterogeneity (Higgins et al., 2003), and the Egger’s test to examine potential publication bias in our findings with a significant test result indicating potential publication bias (Radua et al., 2014).

## Results

The literature search yielded 752 publications in the databases. Based on our inclusion and exclusion criteria, 34 studies (comprising 883 healthy participants) were ultimately identified as suitable for the current meta-analysis, including 26 datasets from sensory unawareness paradigms (733 healthy participants) and 8 datasets from attentional unawareness paradigms (150 healthy participants). Among the included studies, 13 studies provided data on the contrast between positive and neutral face stimuli and 30 studies provided data on the contrast between negative and neutral face stimuli. Information about sample characteristics and the experimental paradigms of the included studies was shown in Table 1.

**Table 1:** Demographic and experimental information of the studies included in this meta-analysis

Dataset	Sample, N	Mean age (Standard Deviation), years	Females, N	Experimental Paradigm	Stimuli and Contrast(s)	Control for low-level confounds
Amting et al., 2010	16	24.9 (2.7)	10	Binocular rivalry	Fearful and disgusted faces vs. neutral faces	Standardization of image luminosity and contrast
Attar et al., 2010	20	26.3 (4.9)	11	Dot motion task	Fearful and happy faces vs. neutral faces	Standardization of image luminosity and spectral energy
Baeken et al., 2012	40	24.4 (5.0)	40	Masking	Negative faces vs. neutral faces	NA
Chen et al., 2015	22	22–25 (age range)	NA	Masking	Fearful, happy and surprised faces vs. neutral faces	Standardization of image luminosity and contrast
Chen et al., 2017	30	23.9 (3.0)	14	Masking	Fearful and angry faces vs. neutral faces	NA
Dannlowski et al., 2007	23	38.7 (12.6)	11	Masking	Angry and sad faces vs. neutral faces	NA
De Martino et al., 2009	15	NA	NA	Attentional blink	Fearful faces vs. neutral faces	NA
Duan et al., 2010	18	23.6 (1.3)	13	Masking	Happy faces vs. neutral faces	NA
Duval et al., 2013	9	23.9 (4.0)	9	Masking	Angry faces vs. neutral faces	NA
Ewbank et al., 2009	22	26.1	NA	Inattention	Fearful and angry faces vs. neutral faces	NA
Faivre et al., 2012	18	18–35 (age range)	12	Crowding	Happy faces vs. neutral faces	Standardization of image luminosity and contrast
Günther et al., 2017	19	22.4 (2.5)	19	Masking	Happy and sad faces vs. neutral faces	NA
Günther et al., 2020	56	26.1 (3.4)	NA	Masking	Fearful faces vs. neutral faces	NA
Holtmann et al., 2013	24	26.8 (5.4)	24	Flanker task	Fearful distractors vs. neutral distractor faces	NA
Ihme et al., 2014	50	23.0 (3.0)	24	Masking	Fearful, angry and happy faces vs. neutral faces	NA
Juruena et al., 2010	10	25.2 (3.2)	3	Masking	Happy and sad faces vs. neutral faces	NA
Kanat et al., 2015	46	NA	NA	Masking	Angry faces vs. neutral faces	NA
Lerner et al., 2012	11	NA	NA	Binocular rivalry	Fearful faces vs. neutral faces	Standardization of image luminosity and contrast
Lichev et al., 2015	46	23.5 (2.7)	23	Masking	Fearful and happy faces vs. neutral faces	NA
Liddell et al., 2005	22	32.0 (13.0)	11	Masking	Fearful faces vs. neutral faces	NA
Lim et al., 2017	19	22.6 (1.3)	10	Masking	Disgusted faces vs. neutral faces	NA
Phillips et al., 2004	8	31.9	0	Masking	Fearful and disgusted faces vs. neutral faces	NA
Pichon et al., 2012	20	25.8 (5.2)	NA	Inattention	Fearful faces vs. neutral faces	NA
Pichon et al., 2016	30	26.4	15	Masking	Fearful faces vs. neutral faces	Standardization of image luminosity
Posner et al., 2011	15	13.4 (1.2)	2	Masking	Fearful faces vs. neutral faces	NA
Rauch et al., 2007	20	24.9 (2.6)	10	Masking	Fearful, angry and happy faces vs. neutral faces	NA
Rosenberg et al., 2020	49	23.3 (2.8)	23	Masking	Happy faces vs. neutral faces	Images normalization
Schulte Holthausen et al., 2016	19	33.7 (11.2)	10	Crowding	Fearful faces vs. other emotions averaged	NA
Suslow et al., 2009	51	28.5 (7.9)	23	Masking	Sad faces vs. neutral faces	Images normalization
Suslow et al., 2019	75	25.8 (3.4)	42	Masking	Fearful and angry faces vs. neutral faces	NA
Tseng et al., 2016	20	15.0 (2.2)	10	Masking	Happy faces vs. neutral faces	NA
Vuilleumier et al., 2001	12	27.7	6	Inattention	Fearful faces vs. neutral faces	NA
Williams et al., 2006	15	35.8 (9.1)	8	Masking	Fearful faces vs. neutral faces	Standardization of image luminosity
Yang et al., 2012	13	NA	NA	Masking	Fearful faces vs. neutral faces	NA

A supplementary discussion on low-level confounds can be found in Supplementary Materials. *Abbreviations*: NA, not available.

**Fig. 2:**
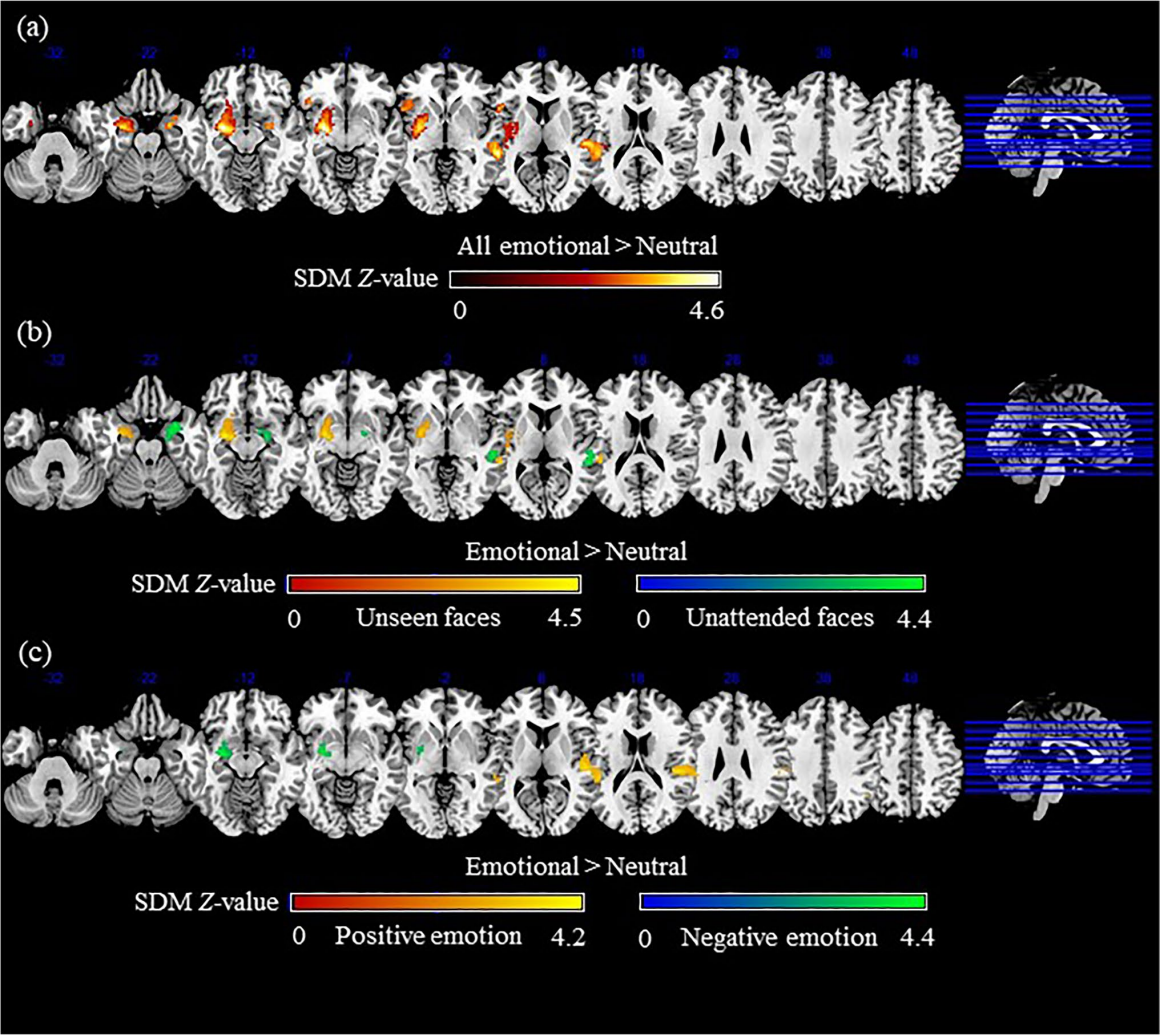
Meta-analyses results of task-evoked activation. Meta-analyses results regarding regional differences of task-evoked activation between (a) all unaware emotional and unaware neutral faces; activation strength is displayed on a black-to-red scale; (b) unseen/unattended emotional and unseen/unattended neutral faces; activation strength is displayed on a red-to-yellow scale for unseen faces and on a blue-to-green scale for unattended faces; (c) unaware positive/negative emotions and unaware neutral faces; activation strength is displayed on a red-to-yellow scale for positive emotions and on a blue-to-green scale for negative emotions. The color bar indicates the maximum and minimum SDM-*Z* values. SDM, seed-based *d* mapping

Across all experimental paradigms, compared to unaware neutral faces, unaware emotional faces showed increased activation in the left striatum (BA 48), extending to the left amygdala (BAs 28, 34, 36), left hippocampus (BAs 28, 34, 35), left parahippocampal gyrus (BAs 28, 36), left rolandic operculum and insula (BA 48), left Heschl’s gyrus (BA 48), left temporal pole (BAs 34, 38) and superior temporal gyrus (STG; BAs 41, 48), left pons and left olfactory cortex (BA 48). Three additional significant clusters consisting of the right STG (BAs 21, 22) and right middle temporal gyrus (MTG; BAs 21, 22), the left inferior frontal gyrus (IFG; BAs 45, 47, 48) extending to the left insula (BAs 47, 48), and the right amygdala (BA 34) extending to the right temporal pole (BA 38) and right parahippocampal gyrus (BA 28) were more strongly activated by unaware emotional compared to neutral faces. Detailed results were shown in Table 2 and Fig. 2a,

**Table 2:** Meta-analysis results regarding regional differences of task-evoked activation between unaware emotional faces and unaware neutral faces

Local Maximum				Cluster		Egger’s test (*p* value)	Jackknife sensitivity	Heterogeneity *I*^*2*^ statistics
Region	Peak MNI coordinate (x, y, z)	SDM-Z value	*p* value	No. of voxels	Breakdown (No. of voxels)			
*All emotional > Neutral*								
L lenticular nucleus, putamen, BA 48	−24,10,-10	3.565	0.000999987	2285	L insula, BA 48 (223) L lenticular nucleus, putamen, BA 48 (210) L rolandic operculum, BA 48 (201) L striatum (198) L amygdala, BA 34 (132) L heschl gyrus, BA 48 (93) L superior temporal gyrus, BA 48 (77) L pons (47) Anterior commissure (47) L inferior network, uncinate fasciculus (44) L superior temporal gyrus, BA 41 (41) L parahippocampal gyrus, BA 28 (39) L amygdala, BA 28 (38) L olfactory cortex, BA 48 (37) L hippocampus (33) L lenticular nucleus, putamen (30) L hippocampus, BA 28 (26) L hippocampus, BA 35 (24) Corpus callosum (22) L inferior network, inferior longitudinal fasciculus (22) L parahippocampal gyrus, BA 36 (19) L inferior network, inferior fronto-occipital fasciculus (18) L amygdala, BA 36 (18) L cortico-spinal projections (17) L temporal pole, superior temporal gyrus, BA 34 (15) L hippocampus, BA 34 (12) L temporal pole, superior temporal gyrus, BA 38 (11) L arcuate network, posterior segment (10)	0.641	34/34	7.2%
R middle temporal gyrus, BA 22	62,-40,0	4.566	0.000999987	794	R superior temporal gyrus, BA 22 (171) R middle temporal gyrus, BA 21 (157) Corpus callosum (134) R middle temporal gyrus, BA 22 (117) R superior temporal gyrus, BA 21 (84) R superior temporal gyrus, BA 48 (68) R superior temporal gyrus, BA 42 (37)	0.276	34/34	4.8%
L inferior frontal gyrus, triangular part, BA 48	−48,16,4	4.288	0.007000029	305	L inferior frontal gyrus, orbital part, BA 47 (64) L inferior frontal gyrus, triangular part, BA 45 (59) L inferior frontal gyrus, opercular part, BA 48 (47) L inferior frontal gyrus, triangular part, BA 47 (44) L insula, BA 48 (24) L inferior frontal gyrus, triangular part, BA 48 (22) L insula, BA 47 (17) L inferior frontal gyrus, triangular part (12)	0.948	29/34	3.1%
R amygdala	24,-4,-22	3.904	0.017000020	227	R amygdala, BA 34 (65) R temporal pole, superior temporal gyrus, BA 38 (18) R parahippocampal gyrus, BA 28 (14) R inferior network, uncinate fasciculus (13) R inferior network, inferior longitudinal fasciculus (11)	0.086	27/34	33.3%
*All emotional < Neutral*								
None								

*BA* Brodmann area, *R* right, *L* left.

A subgroup analysis on datasets from sensory unawareness paradigms showed that, compared to unseen neutral faces, unseen emotional faces elicited increased activation in the left striatum (BA 48), extending to the left amygdala (BAs 28, 34, 36), left hippocampus (BAs 28, 34, 35), left rolandic operculum and insula (BA 48), left Heschl’s gyrus (BA 48) and STG (BAs 41, 48), left pons and left olfactory cortex (BA 48). Another significant cluster consisting of the right MTG (BAs 21, 22) extending to the right STG (BAs 21, 22) was also more strongly activated by unseen emotional compared to neutral faces.

A subgroup analysis on datasets from attentional unawareness paradigms showed that unattended emotional faces elicited increased activation of the right temporal pole (BA 38), extending to the right amygdala (BAs 28, 34), right hippocampus (BA 34), right parahippocampal gyrus (BA 28) and right striatum, compared to unattended neutral faces. Additionally, unattended emotional faces showed increased activation of the right MTG and STG (BAs 21, 22), compared to unattended neutral faces. Detailed results were presented in Table 3 and Fig. 2b.

**Table 3:** Meta-analysis results regarding regional differences of task-evoked activation between unaware emotional faces and unaware neutral faces in studies using masking or binocular rivalry (unseen stimuli) and studies using inattention paradigms (unattended stimuli)

Local Maximum				Cluster		Egger’s test (*p* value)	Jackknife sensitivity	Heterogeneity *I*^*2*^ statistics
Region	Peak MNI coordinate (x, y, z)	SDM-Z value	*p* value	No. of voxels	Breakdown (No. of voxels)			
*Unseen emotional > Unseen neutral*								
L striatum	−24,-8,-10	4.429	0.001999974	1177	L lenticular nucleus, putamen, BA 48 (144) L amygdala, BA 34 (127) L striatum (118) L rolandic operculum, BA 48 (104) L heschl gyrus, BA 48 (52) L superior temporal gyrus, BA 48 (50) L insula, BA 48 (49) Anterior commissure (44) L olfactory cortex, BA 48 (32) L pons (30) L superior temporal gyrus, BA 41 (22) L lenticular nucleus, putamen (20) L hippocampus (19) L amygdala, BA 28 (16) L hippocampus, BA 35 (15) L amygdala, BA 36 (13) L hippocampus, BA 28 (13) L inferior network, uncinate fasciculus (11) L inferior network, inferior longitudinal fasciculus (10) L hippocampus, BA 34 (10)	0.448	26/26	6.9%
R middle temporal gyrus, BA 22	62,-40,0	3.886	0.023000002	135	R middle temporal gyrus, BA 22 (54) R middle temporal gyrus, BA 21 (42) R superior temporal gyrus, BA 22 (21) R superior temporal gyrus, BA 21 (18)	0.254	19/26	12.5%
*Unseen emotional < Unseen neutral*								
None								
*Unattended emotional > Unattended neutral*								
R temporal pole, superior temporal gyrus, BA 38	30,4,−24	4.345	0.000999987	479	R amygdala, BA 34 (93) R temporal pole, superior temporal gyrus, BA 38 (32) R amygdala, BA 36 (26) R inferior network, inferior longitudinal fasciculus (25) R inferior network, uncinate fasciculus (24) R hippocampus (19) R parahippocampal gyrus, BA 28 (18) R amygdala, BA 28 (12) R striatum (11) R cortico-spinal projections (10) R hippocampus, BA 34 (10)	0.374	7/8	6.3%
R middle temporal gyrus, BA 21	54,-32,0	4.145	0.001999974	293	R middle temporal gyrus, BA 21 (69) R superior temporal gyrus, BA 22 (58) R superior temporal gyrus, BA 21 (37) R middle temporal gyrus, BA 22 (30)	0.632	6/8	8.2%
*Unattended emotional < Unattended neutral*								
None								

*BA* Brodmann area, *R* right, *L* left.

A subgroup analysis on datasets on positive emotions showed that, unaware faces with positive emotions (e.g., happy faces) elicited increased activation of the right Heschl’s gyrus (BA 48), extending to the right rolandic operculum (BA 48), right STG (BAs 22, 42, 48), right MTG (BAs 21, 22) and right supramarginal gyrus (BAs 42, 48), compared to unaware neutral faces. A subgroup analysis on datasets on negative emotions showed that unaware negative faces (e.g., fearful and angry faces) elicited increased activation in the left striatum (BA 48), extending to the left amygdala (BA 34) and left pons, compared to unaware neutral faces. Detailed results were presented in Table 4 and Fig. 2c.

**Table 4:** Meta-analysis results regarding regional differences of task-evoked activation between unaware faces with positive or negative emotions and unaware neutral faces

Local Maximum				Cluster		Egger’s test (*p* value)	Jackknife sensitivity	Heterogeneity *I*^*2*^ statistics
Region	Peak MNI coordinate (x, y, z)	SDM-Z value	*p* value	No. of voxels	Breakdown (No. of voxels)			
*Positive > Neutral*								
R heschl gyrus, BA 48	44,-26,14	4.120	0.000999987	933	R rolandic operculum, BA 48 (183) R superior temporal gyrus, BA 22 (122) R superior temporal gyrus, BA 48 (110) R heschl gyrus, BA 48 (103) R superior temporal gyrus, BA 42 (95) Corpus callosum (69) R middle temporal gyrus, BA 22 (64) R supramarginal gyrus, BA 48 (61) R middle temporal gyrus, BA 21 (36) R superior temporal gyrus, BA 21 (26) R superior temporal gyrus (24) R supramarginal gyrus, BA 42 (14)	0.892	11/13	7.9%
*Positive < Neutral*								
None								
*Negative > Neutral*								
L striatum	-24,-8,-10	4.317	0.009000003	325	L amygdala, BA 34 (59) L lenticular nucleus, putamen, BA 48 (46) L striatum (42) Anterior commissure (35) L pons (18)	0.382	30/30	10.0%
*Negative < Neutral*								
None								

*BA* Brodmann area, *R* right, *L* left.

Emotional faces did not show any significant reduced activation, compared to neutral faces, in any of the analyses conducted.

The findings described above remained largely unchanged under the jackknife sensitivity analysis, indicating high robustness of the results (Tables 2, 3, 4). The *I*^*2*^ statistics from the heterogeneity analysis showed that all reported regions had low unexplained between-study variabilities (i.e., *I*^*2*^ < 50%). The Egger’s tests showed no evidence of publication bias for all the reported regions.

## Discussion

### Unaware emotional vs neutral faces across paradigms

Across all experimental paradigms, unaware emotional faces could be distinguished from unaware neutral faces by engaging subcortical regions (e.g., amygdala and striatum) and limbic areas (e.g., hippocampus and parahippocampal gyrus). This finding is consistent with the extensive literature on emotion processing. Previous research has found that emotional information including fearful, angry and happy faces is associated with stronger activation of the amygdala and hippocampus, compared to a neutral counterpart, during nonconscious visual processing (for reviews see Diano et al., 2017; Phelps & LeDoux, 2005). Similarly, in studies of persons with blindsight, emotional expressions were found to activate the amygdala even when these patients were cortically blind and thus unaware of the presence of visual stimuli (Pegna et al., 2005; Tamietto & De Gelder, 2010). As mentioned in the Introduction, a subcortical colliculus-pulvinar-amygdala pathway has been suggested to be necessary for unconscious emotion processing (Morris et al., 1999; Kragel et al., 2021; LeDoux, 1998, 2000; Méndez-Bértolo et al., 2016). This is particularly striking for patients who are deprived of primary visual cortices but show affective blindsight (Morris et al., 2001). For these patients, it has been posited that emotional and threat-related signals may reach the amygdala through this alternate route, bypassing the geniculo-striate path, which enables reflexive responses to potential threats in our environment (Morris et al., 1999).

However, the existence of such a pathway has been contested. Indeed, some researchers argue that cortical responses rather than subcortical pathways may account for unconscious emotion processing (Cauchoix & Crouzet, 2013; Pessoa & Adolphs, 2010; Palermo & Rhodes, 2007; Sanchez-Lopez et al., 2020). Additionally, it has been argued that intact projections from the lateral geniculate nucleus to extrastriate areas, which also bypasses V1, may subserve blindsight in humans (Ajina & Bridge, 2017; Ajina et al., 2015; Smits et al., 2019). Thus, it remains open to debate whether and to what extent unconscious emotion processing is subserved by the subcortical pathway that targets the amygdala. Although our meta-analysis reveals that the amygdala is more strongly activated for emotional relative to neutral faces when visual awareness is restricted, the pathways leading to this activation cannot be determined without further investigations.

Interestingly, the amygdala and the striatum have been found to be actively involved in the striatal dopaminergic system, which has been implicated in the processing of negative emotions (Badgaiyan, 2010; Sprengelmeyer et al., 2003). For example, increased release of dopamine at the dorsal striatum was found in response to negative emotions (Badgaiyan, 2010).

Taken together, in line with the findings reported in blindsight patients (e.g., Pegna et al., 2005; Tamietto & De Gelder, 2010), our current meta-analysis shows that, in the absence of visual awareness, emotional relative to neutral faces increase activation of subcortical regions as well as limbic areas in healthy individuals, potentially reflecting a stronger neural representation of emotional expressions.

Our meta-analysis also revealed that cortical regions including the IFG, insula, STG, MTG and temporal pole were more strongly activated in response to unaware emotional compared to neutral faces. The IFG and insula are important for the integration of external information and internal bodily experience (e.g., emotional arousal; Terasawa et al., 2013) and show increased activity during conscious emotional experience (Miller & Cohen, 2001; Vytal & Hamann, 2010). Similarly, regions in the temporal cortex have been found to play a crucial role in emotion recognition (Fried et al., 1997) and the identification of emotional features (Adolphs, 2002). Fearful and angry expressions, for example, were found to be associated with stronger activation of the MTG and several adjacent regions including the insula, compared to neutral faces, when presented supraliminally (Goghari et al., 2011). Our results confirm the roles of these cortical regions in emotion processing by showing that, even when visual awareness is highly restricted, the IFG, insula and regions in the temporal cortex can be significantly activated by emotional faces.

However, there have been several claims that the activation of these regions, in particular the amygdala, do not differ between unconsciously processed emotional and neutral faces (Pessoa et al., 2002, 2005). The existing contradictory results could be partially due to different experimental tasks. For example, in inattention tasks, one major difference is whether the facial emotions or the faces themselves were being attended. In some experiments, inattention towards the face stimuli was implemented by requiring participants to respond to non-emotional aspects of the faces (e.g., gender or age of the faces; Anderson et al., 2003; Habel et al., 2007). This is different from inattention implemented by asking participants to ignore the faces altogether and attend to images presented elsewhere (e.g., Vuilleumier et al., 2001), or overlapping images presented in the same spatial location (e.g., moving dots; Attar et al., 2010). In the current meta-analysis, we included studies where the faces were not attended at all for the inattention paradigms. With this stricter inclusion criterion in place, our current meta-analytic results reconcile the mixed findings with robust quantitative evidence showing that the limbic areas and contiguous cortical regions are indeed more strongly activated by emotional faces, compared to neutral faces, when they are not consciously processed.

Moreover, across different inattention paradigms, other attention-related factors may vary. Specifically, the processing of unattended emotional faces has been found to be sensitive to participants’ attentional load and task goals (Pessoa et al., 2002, 2005). The inconsistencies between reporting increased neural responses for emotional relative to neutral faces in some studies (e.g., Vuilleumier et al., 2001) and the absence of such effects in other studies (e.g., Pessoa et al., 2002) could thus be due to differences in the control or manipulation of participants’ attention. Further research can aim to systematically examine whether and how the strength of enhanced neural responses to unattended emotional faces changes as participants’ attentional conditions vary.

### Comparing sensory and attentional unawareness

Importantly in our examination of previous studies, different experimental paradigms showed different activation patterns when comparing unaware emotional to neutral faces. We found that attentional unawareness paradigms showed a right lateralization of activation. Specifically, inattention paradigms revealed stronger activity in the right amygdala and right temporal pole for unattended emotional compared to neutral faces. However, sensory unawareness paradigms (masking or binocular rivalry) revealed increased activations of the left striatum, left amygdala and right MTG by unseen emotional compared to neutral faces.

Right hemisphere dominance has been widely investigated in the literature on face processing (e.g., face identity recognition; Vuilleumier et al., 2003 and emotion processing; Demaree et al., 2005) and attention (e.g., De Schotten et al., 2011; Shulman et al., 2010; Weintraub & Mesulam, 1987). Studies on patients with unilateral spatial neglect have consistently shown that right parietal lesions are associated with more severe spatial neglect symptoms compared to left parietal lesions (for a review see Parton et al., 2004). In healthy individuals, the right visual cortex and its connections with the right amygdala are also implicated in the processing of emotional faces (Noesselt et al., 2005). Specifically, using a bilateral presentation of faces, Noesselt et al. (2005) found that participants’ right visual cortex showed enhanced hemodynamic responses and increased connectivity with the right amygdala after the viewing of a fearful face, but not a neutral face, presented in the left hemifield. Consistent with these observations, our results show that for healthy individuals, right lateralized brain regions including the right amygdala are more strongly activated by unattended emotional relative to neutral faces, during inattention.

Comparisons of left and right hemianopics might provide further insights into the right lateralized activation by unattended emotional faces reported here (e.g., Bertini et al., 2013, 2018, 2019). In a series of experiments, emotional faces were presented supraliminally to the lesioned patients while the patients were required to respond either to the emotion of the faces presented in their blind visual field alone or to information concurrently presented in their intact visual field (for a review see Làdavas & Bertini, 2021). It was found that, while both left- and right-lesioned hemianopic patients could not detect stimuli presented alone in the blind visual field, patients with lesions to the left visual cortices tend to show improved performance at discriminating the emotion of faces in the intact field when a fearful face was concurrently shown in their blind field (e.g., Bertini et al., 2013). However, patients with right hemispheric lesions did not show such an implicit processing bias for fear-related signals in their blind field. It was therefore suggested that the right hemisphere is key to the unconscious processing of fearful faces (Làdavas & Bertini, 2021). As pointed out by Làdavas and Bertini (2021), when presented concurrently with task-relevant information, the task-irrelevant emotional faces in the blind visual field can provide ambiguous information outside the accessible visual field. As attention has been deployed to parts of the visual display (i.e., intact visual field), information across the overall display may be able to access some, albeit limited, attentional resources. Consequently, unattended emotional faces could be processed possibly by engaging the right attention system, in left-lesioned hemianopic patients. In contrast, when information was presented only within the blind visual field of the patients, or when patients had right-hemispheric lesions, attention mechanisms were likely not activated at all, which could explain why emotional faces were not distinguished from neutral faces in both cases (Làdavas & Bertini, 2021). Here, our results show that a large right-lateralized neural network can be more strongly activated by unattended emotional relative to neutral faces in healthy individuals. However, emotional faces rendered unseen by masking or binocular rivalry seem unable to engage the right hemisphere system related to attention processes.

It should be noted that the number of included inattention studies was small (i.e., eight). This is due to the rather stringent inclusion criteria we used to obtain less biased meta-analytic results. Indeed, the results across all analyses were robust as indicated by high Jackknife sensitivity scores, low *I*^*2*^ statistics and non-significant Egger’s test results. Further research is needed nevertheless to provide more insights into the neural fate of unattended emotional faces compared to unseen stimuli. Future research on unconscious emotion processing should also validate their manipulations of unawareness, especially in inattention studies.

Additionally, the distinction between task-irrelevant faces and task-irrelevant emotions should be regarded as crucial in the examination of the neural fate of unattended emotional faces. A previous meta-analysis on the implicit processing of emotional faces treated task-irrelevant faces and task-irrelevant emotions as a single category, and revealed stronger activation of higher-level frontal (i.e., IFG) and parietal regions (i.e., precuneus) for emotional faces, compared to neutral ones (Shi et al., 2013). However, our current results did not show activation of these regions, presumably because we limited the investigation to unattended faces. It is likely that the activation of higher-level cortical regions was specific to the attended emotional information about the faces (Shi et al., 2013). It is thus clear that minor variations in defining inattention can result in largely different meta-analytic results.

### Comparing positive and negative emotions

In the current meta-analysis, we also identified different activation patterns for positive and negative emotions. Compared to unaware neutral faces, unaware positive emotions were associated with stronger activation of the temporal and parietal cortices whereas unaware negative emotions elicited increased activation of the striatum and amygdala. The involvement of subcortical regions in the unconscious processing of negative emotions (e.g., fear, anger) has been well documented in the literature, as described above. Because negative emotions can act as informative cues about our environment, oftentimes indicating potential danger, a processing bias arises towards such stimuli (Öhman & Mineka, 2001), which as explained above, may be prioritized for processing through the subcortical pathway (Tamietto & De Gelder, 2010).

By contrast, positive emotions like happy faces may not necessitate a fast relay of information as they tend not to be associated with threat, or require quick responses. As suggested by Xu et al. (2021), while negative emotions are likely processed rapidly through the subcortical path in addition to the cortical route, positive emotions like happy faces may be mainly processed via the latter. In the absence of visual awareness, subcortical regions may be therefore unable to efficiently distinguish neutral faces from positive ones. In line with this suggestion, our meta-analytic results show that the unconscious processing of positive emotions involves several temporal and parietal regions, consistent with previous research where increased activity in the temporal and parietal lobes were found during the processing of happy expressions (for a review see Machado & Cantilino, 2017).

## Conclusion

The current meta-analysis shows that unconsciously processed emotional faces elicit stronger activation of the limbic system, subcortical areas (i.e., striatum) and several cortical regions (i.e., IFG, insula and the temporal lobe), compared to neutral faces. Crucially, a right hemisphere dominance was found for the unconscious processing of emotional faces in attentional unawareness but not sensory unawareness. Additionally, in the absence of visual awareness, positive emotions were found to be associated with stronger activity in temporal and parietal cortices, whereas negative emotions were found to elicit stronger activation of subcortical regions including the amygdala and striatum, when compared to neutral faces. These findings indicate variations in patterns of activity in different conditions that reflect unconscious processing of emotions. Future studies could address these differences in a more systematic manner.

## Supplementary information

ESM 1 (DOCX 43 kb)

ESM 2 (DOCX 25 kb)

## Data Availability

Not applicable.
