# Supplementary material for: Neural activities during the Processing of unattended and unseen emotional faces: a voxel-wise Meta-analysis
Source: Brain Imaging Behav. 2022 Jun 23;16(5):2426–43. doi: 10.1007/s11682-022-00697-8 (PMC9581832; doi:10.1007/s11682-022-00697-8)
Supplement: Supplementary file 1 — (DOCX 43 kb) [file 11682_2022_697_MOESM1_ESM.docx]

**Neural Activities during the Processing of Unattended and Unseen Emotional Faces: A Voxel-wise Meta-analysis**

**Supplementary Contents**

**Table S1.** Quality assessment checklist.

**Table S2.** Image acquisition details and quality assessment scores of task-based fMRI studies included in the meta-analyses.

**Table S3.** Meta-analysis results regarding regional differences of task-evoked activation between unaware emotional faces and unaware neutral faces for the true-unawareness subgroup.

**Supplementary Discussion.** On low-level confounds

**Supplementary References.** Articles included in the meta-analyses.

Table S1. Quality assessment checklist.

| Items (score 0/0.5/1 per item; total score out of 7) |
| --- |
| Category I: Participants |
| 1. Healthy participants were evaluated prospectively, psychiatric and medical illness were excluded. |
| Category II: Methods for image acquisition and analysis |
| 1. Whole brain analysis was automated with no a prior regional selection. 2. Coordinates reported in a standard space. 3. The imaging technique used was clearly described so that it could be reproduced. 4. Measurements were clearly described so that they could be reproduced. |
| Category III: Results and conclusions |
| 1. Statistical parameters for significant and important nonsignificant differences were provided. 2. Conclusions were consistent with the results obtained and the limitations were discussed. |

*Note*: When criteria were partially met, 0.5 points were awarded.

Table S2. Image acquisition details and quality assessment scores of task-based fMRI studies included in the meta-analyses.

| Dataset | Data Analysis | | |  | Image Acquisition | | | Source of Coordinates Included | Quality Assessment |
| --- | --- | --- | --- | --- | --- | --- | --- | --- | --- |
|  | Correction for Multiple Comparisons | Threshold (Voxel-level) | Threshold (Cluster-level) |  | Scanner | Software | Coordinate Space |  | Total Scores/7 |
| Amting et al., (2010)  Attar et al., (2010) | Uncorrected  FWE corrected | NA  NA | *p* < .001  *p* < .001 |  | 3T  3T | AFNI  SPM | MNI  MNI | Main effect of unperceived emotion  Table 1. | 6  6.5 |
| Baeken et al., (2012)  Chen et al. (2015)  Chen et al. (2017)  Dannlowski et al. (2007)  De Martino et al. (2009)  Duan et al. (2010)  Duval et al. (2013)  Ewbank et al. (2009)  Faivre et al. (2012)  Gunther et al. (2017)  Gunther et al. (2020)  Holtmann et al. (2013)  Ihme et al. (2014)  Juruena et al. (2010)  Kanat et al. (2015)  Lerner et al. (2012)  Lichev et al. (2015)  Liddell et al. (2005)  Lim et al. (2017)  Phillips et al. (2004)  Pichon et al. (2012)  Pichon et al. (2016)  Posner et al. (2011)  Rauch et al. (2007)  Rosenberg et al. (2020)  Schulte Holthausen et al. (2016)  Suslow et al. (2009)  Suslow et al. (2019)  Tseng et al. (2016)  Vuilleumier et al. (2001)  Williams et al. (2006) | corrected  corrected  FWE corrected  Uncorrected  FWE corrected  Uncorrected  uncorrected  uncorrected  uncorrected  Uncorrected  FWE corrected  Uncorrected  FWE corrected  NA  uncorrected  corrected  uncorrected  uncorrected  corrected  unspecified  FEW corrected  Uncorrected  Corrected  FDR  corrected  FWE corrected  FWE corrected  FDR corrected  FWE corrected  Uncorrected  Corrected  Unspecified | NA  NA  *p* < .05  *p* < .001  NA  *p<* .001  NA  NA  NA  NA  *p <* .001 (uncorrected)  NA  NA  NA  NA  NA  NA  NA  NA  *p* < .005  NA  NA  *p* < .001  NA  *p* < .001  NA  NA  NA  *p* < .005  *p* < .05  NA | *p* < .05  *p <* .05  *p <* .001 (uncorrected)  NA  NA  *p* < .05  NA  *p<* .005  *p<* .001  *p<* .001  *p* < .001  *p <* .05  *p* < .001  *p* < .05  NA  *p* < .001  *p* < .05  *p* < .001  *p* < .001  *p* < .05  NA  *p* < .05  *p* < .001  (uncorrected)  *p* < .001  NA  *p* < .05  *p* < .05  *p* < .05  *p* < .05  *p* < .05  NA  NA  *p* < .001 |  | 1.5T  3T  3T  3T  1.5T  3T  3T  3T  3T  3T  3T  3T  3T  1.5T  3T  3T  3T  1.5T  3T  1.5T  3T  3T  3T  3T  3T  3T  3T  3T  3T  2T  1.5T | SPM  SPM  SPM  SPM  SPM  SPM  Brain Voyager  SPM  SPM  SPM  SPM  SPM  SPM  XBAM  SPM  Brain  Voyager  SPM  SPM  FSL  Others  SPM  SPM  SPM  SPM  SPM  SPM  SPM  SPM  Others  SPM  SPM | MNI  MNI  NA  MNI  MNI  MNI  TAL  MNI  MNI  MNI  MNI  MNI  MNI  TAL  MNI  TAL  MNI  MNI  MNI  TAL  MNI  MNI  MNI  MNI  MNI  MNI  MNI  MNI  TAL  MNI  MNI | In-text Positive versus negative emotional priming Table 4.  Table 1.  Table S2.  Table 2.  Figure 3.  Table 1.  Result  Table 4.  Table 1.  3.2.2. Group comparisons when controlling for social anxiety: whole-brain analyses + Table S1. (PE)  3.3.2. Unaware group  Table 4.  Table 4.  Results  Whole brain analysis + Table S4.  fMRI results  Main effects of masked emotion faces on brain activation: whole-brain analysis  Table 1.  Contrasting neural processing of disgust and neutral priming – presentation and decision.  Table 1.  fMRI result Table 1.  Table 2.  Table 2.  Table 2.  Neuroimaging results  Table 2.  Table 1.  Table 2. Table 3.  Table 2.  Effects of Fear Independent of Attentional Condition  Effect of Awareness on Whole-Brain Activity | 7  6.5  6.5  5.5  7  6  7  7  7  7  7  7  7  6.5  6.5  6.5  6.5  6.5  6.5  7  7  7  7  7  7  6.5  7  7  6  6.5  7 |
| Yang et al. (2012) | corrected | *p* < .02 | NA |  | 3T | AFNI | TAL | Table 1. | 6.5 |

*Abbreviations*: FDR, false discovery rate; FWE, family-wise error; FSL, FMRIB’s Software Library; SPM, Statistical Parametric Mapping; MNI, Montreal Neurological Institute; TAL, Talairach space; NA, not available.

We collected information on whether participants’ unawareness of stimuli was confirmed in the individual studies, specifically by using chance-level performance at a face detection task and/or subjective report of not seeing any face stimuli as indicators of unawareness. Twenty-four out of 34 studies provided such measurements and analyzed data only from participants who reported no awareness of the stimuli. We then ran an additional analysis using data from this subgroup, across all experimental paradigms. The results from this additional analysis remained largely the same as the analysis using all available datasets. Results from this subgroup analysis are presented in Table S3 below. To provide a more comprehensive examinations of the effects of interest, we reported the results from the analyses using all 34 available datasets in the manuscript.

**Table S3.** Meta-analysis results regarding regional differences of task-evoked activation between unaware emotional faces and unaware neutral faces for the true-unawareness subgroup.

| Local Maximum |  |  |  |  | Cluster | | Egger’s test  (*p* value) | Jackknife sensitivity | Heterogeneity *I^2^* statistics |
| --- | --- | --- | --- | --- | --- | --- | --- | --- | --- |
| Region | Peak MNI coordinate  (x, y, z) | SDM-Z value | *p* value |  | No. of voxels | Breakdown (No. of voxels) |  |  |  |
| *All emotional > Neutral* |  |  |  |  |  |  |  |  |  |
| R temporal pole, superior temporal gyrus | 34,6,-20 | 3.553 | 0.013999999 |  | 209 | R amygdala (100)  R temporal pole, superior temporal gyrus (27)  R parahippocampal gyrus (18)  R inferior network (24)  R hippocampus (9) | 0.221 | 18/24 | 13.9% |
| L lenticular nucleus, putamen, BA 48 | -28,-6,-24 | 3.449 | 0.015999973 |  | 200 | L amygdala (74)  L striatum/lenticular nucleus, putamen (50)  L hippocampus (18)  L parahippocampal gyrus (3) | 0.219 | 18/24 | 25.2% |
|  |  |  |  |  |  |  |  |  |  |
| *All emotional < Neutral* |  |  |  |  |  |  |  |  |  |
| None |  |  |  |  |  |  |  |  |  |

*Abbreviations*: BA, Brodmann area; R, Right; L, left.

**Supplementary Discussion**

On low-level confounds

As rightfully pointed out by a reviewer, it is crucial for research in face processing to control for low-level differences across face stimuli. While the literature has documented the relevance of emotional expression during unconscious face processing when low-level features were controlled for (Vetter et al., 2019, Elife; Yang & Yeh, 2018, Psychonomic bulletin & review), some researchers have provided behavioral evidence that such a processing bias for emotional expressions, fear in particular, was due to low-level features rather than higher-level meaning of the emotion (Gray et al., 2013, Emotion; Hedger et al., 2015, Journal of Experimental Psychology: Human Perception and Performance). In the current meta-analysis, only nine out of 34 included studies explicitly stated that they matched the low-level features across face stimuli. It would be desirable if future fMRI research on relevant topics can control for low-level differences across face stimuli, for example, by including a control condition where the same face stimuli are presented upside-down (e.g., Gray et al., 2013).

**Supplementary References**

Amting, J. M., Greening, S. G., & Mitchell, D. G. (2010). Multiple mechanisms of consciousness: the neural correlates of emotional awareness. *Journal of Neuroscience*, *30*(30), 10039-10047. <https://doi.org/10.1523/JNEUROSCI.6434-09.2010>

Attar, C. H., Müller, M. M., Andersen, S. K., Büchel, C., & Rose, M. (2010). Emotional processing in a salient motion context: integration of motion and emotion in both V5/hMT+ and the amygdala. *Journal of Neuroscience*, *30*(15), 5204-5210. <https://doi.org/10.1523/JNEUROSCI.5029-09.2010>

Baeken, C., De Raedt, R., Van Schuerbeek, P., De Mey, J., Bossuyt, A., & Luypaert, R. (2012). The influence of emotional priming on the neural substrates of memory: A prospective fMRI study using portrait art stimuli. *NeuroImage*, 61(4), 876-883. <https://doi.org/10.1016/j.neuroimage.2012.03.043>

Chen, C., Hu, C. H., & Cheng, Y. (2017). Mismatch negativity (MMN) stands at the crossroads between explicit and implicit emotional processing. *Human brain mapping*, *38*(1), 140-150. <https://doi.org/10.1002/hbm.23349>

Chen, P. H. A., Whalen, P. J., Freeman, J. B., Taylor, J. M., & Heatherton, T. F. (2015). Brain reward activity to masked in-group smiling faces predicts friendship development. *Social psychological and personality science*, *6*(4), 415-421. <https://doi.org/10.1177/1948550614566093>

Dannlowski, U., Ohrmann, P., Bauer, J., Kugel, H., Arolt, V., Heindel, W., & Suslow, T. (2007). Amygdala reactivity predicts automatic negative evaluations for facial emotions. *Psychiatry Research: Neuroimaging*, *154*(1), 13-20. https://doi.org/10.1016/j.pscychresns.2006.05.005

De Martino, B., Kalisch, R., Rees, G., & Dolan, R. J. (2009). Enhanced processing of threat stimuli under limited attentional resources. *Cerebral Cortex*, *19*(1), 127-133. <https://doi.org/10.1093/cercor/bhn062>

Duan, X., Dai, Q., Gong, Q., & Chen, H. (2010). Neural mechanism of unconscious perception of surprised facial expression. *Neuroimage*, *52*(1), 401-407. <https://doi.org/10.1016/j.neuroimage.2010.04.021>

Duval, E. R., Hale, L. R., Liberzon, I., Lepping, R., Powell, J. N., Filion, D. L., & Savage, C. R. (2013). Anterior cingulate cortex involvement in subclinical social anxiety. *Psychiatry Research: Neuroimaging*, *214*(3), 459-461. <https://doi.org/10.1016/j.pscychresns.2013.09.005>

Ewbank, M. P., Lawrence, A. D., Passamonti, L., Keane, J., Peers, P. V., & Calder, A. J. (2009). Anxiety predicts a differential neural response to attended and unattended facial signals of anger and fear. *Neuroimage*, *44*(3), 1144-1151. <https://doi.org/10.1016/j.neuroimage.2008.09.056>

Faivre, N., Charron, S., Roux, P., Lehéricy, S., & Kouider, S. (2012). Nonconscious emotional processing involves distinct neural pathways for pictures and videos. *Neuropsychologia*, *50*(14), 3736-3744. <https://doi.org/10.1016/j.neuropsychologia.2012.10.025>

Günther, V., Hußlack, A., Weil, A. S., Bujanow, A., Henkelmann, J., Kersting, A., ... & Suslow, T. (2020). Individual differences in anxiety and automatic amygdala response to fearful faces: A replication and extension of Etkin et al.(2004). *NeuroImage: Clinical*, *28*, 102441. https://doi.org/10.1016/j.nicl.2020.102441

Günther, V., Zimmer, J., Kersting, A., Hoffmann, K. T., Lobsien, D., & Suslow, T. (2017). Automatic processing of emotional facial expressions as a function of social anhedonia. *Psychiatry Research: Neuroimaging*, *270*, 46-53. <https://doi.org/10.1016/j.pscychresns.2017.10.002>

Holtmann, J., Herbort, M. C., Wüstenberg, T., Soch, J., Richter, S., Walter, H., ... & Schott, B. H. (2013). Trait anxiety modulates fronto-limbic processing of emotional interference in borderline personality disorder. *Frontiers in human neuroscience*, *7*, 54. <https://doi.org/10.3389/fnhum.2013.00054>

Ihme, K., Sacher, J., Lichev, V., Rosenberg, N., Kugel, H., Rufer, M., ... & Suslow, T. (2014). Alexithymic features and the labeling of brief emotional facial expressions–An fMRI study. *Neuropsychologia*, *64*, 289-299. <https://doi.org/10.1016/j.neuropsychologia.2014.09.044>

Juruena, M. F., Giampietro, V. P., Smith, S. D., Surguladze, S. A., Dalton, J. A., Benson, P. J., ... & Fu, C. H. (2010). Amygdala activation to masked happy facial expressions. *Journal of the International Neuropsychological Society*, *16*(2), 383-387. <https://doi.org/10.1017/S1355617709991172>

Kanat, M., Heinrichs, M., Schwarzwald, R., & Domes, G. (2015). Oxytocin attenuates neural reactivity to masked threat cues from the eyes. *Neuropsychopharmacology*, *40*(2), 287-295. <https://doi.org/10.1038/npp.2014.183>

Lerner, Y., Singer, N., Gonen, T., Weintraub, Y., Cohen, O., Rubin, N., ... & Hendler, T. (2012). Feeling without seeing? Engagement of ventral, but not dorsal, amygdala during unaware exposure to emotional faces. *Journal of Cognitive Neuroscience*, *24*(3), 531-542. <https://doi.org/10.1162/jocn_a_00165>

Lichev, V., Sacher, J., Ihme, K., Rosenberg, N., Quirin, M., Lepsien, J., ... & Suslow, T. (2015). Automatic emotion processing as a function of trait emotional awareness: an fMRI study. *Social Cognitive and Affective Neuroscience*, *10*(5), 680-689. <https://doi.org/10.1093/scan/nsu104>

Liddell, B. J., Brown, K. J., Kemp, A. H., Barton, M. J., Das, P., Peduto, A., ... & Williams, L. M. (2005). A direct brainstem–amygdala–cortical ‘alarm’system for subliminal signals of fear. *Neuroimage*, *24*(1), 235-243. <https://doi.org/10.1016/j.neuroimage.2004.08.016>

Lim, J., Kurnianingsih, Y. A., Ong, H. H., & O’Dhaniel, A. (2017). Moral judgment modulation by disgust priming via altered fronto-temporal functional connectivity. *Scientific reports*, *7*(1), 1-14. <https://doi.org/10.1038/s41598-017-11147-7>

Phillips, M. L., Williams, L. M., Heining, M., Herba, C. M., Russell, T., Andrew, C., ... & Gray, J. A. (2004). Differential neural responses to overt and covert presentations of facial expressions of fear and disgust. *Neuroimage*, *21*(4), 1484-1496. https://doi.org/10.1016/j.neuroimage.2003.12.013

Pichon, S., Guex, R., & Vuilleumier, P. (2016). Influence of temporal expectations on response priming by subliminal faces. *Plos one*, *11*(10), e0164613. https://doi.org/10.1371/journal.pone.0164613

Pichon, S., Rieger, S. W., & Vuilleumier, P. (2012). Persistent affective biases in human amygdala response following implicit priming with negative emotion concepts. *NeuroImage*, *62*(3), 1610-1621. https://doi.org/10.1016/j.neuroimage.2012.06.004

Posner, J., Nagel, B. J., Maia, T. V., Mechling, A., Oh, M., Wang, Z., & Peterson, B. S. (2011). Abnormal amygdalar activation and connectivity in adolescents with attention-deficit/hyperactivity disorder. *Journal of the American Academy of Child & Adolescent Psychiatry*, *50*(8), 828-837. <https://doi.org/10.1016/j.jaac.2011.05.010>

Rauch, A. V., Ohrmann, P., Bauer, J., Kugel, H., Engelien, A., Arolt, V., ... & Suslow, T. (2007). Cognitive coping style modulates neural responses to emotional faces in healthy humans: a 3-T FMRI study. *Cerebral Cortex*, *17*(11), 2526-2535. <https://doi.org/10.1093/cercor/bhl158>

Rosenberg, N., Ihme, K., Lichev, V., Sacher, J., Rufer, M., Grabe, H. J., ... & Suslow, T. (2020). Alexithymia and automatic processing of facial emotions: behavioral and neural findings. *BMC neuroscience*, *21*, 1-13. https://doi.org/10.1186/s12868-020-00572-6

Schulte Holthausen, B., Habel, U., Kellermann, T., Schelenz, P. D., Schneider, F., Christopher Edgar, J., ... & Regenbogen, C. (2016). Task-irrelevant fear enhances amygdala-FFG inhibition and decreases subsequent face processing. *Social cognitive and affective neuroscience*, *11*(9), 1440-1448. <https://doi.org/10.1093/scan/nsw054>

Suslow, T., Hußlack, A., Bujanow, A., Henkelmann, J., Kersting, A., Hoffmann, K. T., ... & Günther, V. (2019). Implicitly and explicitly assessed anxiety: no relationships with recognition of and brain response to facial emotions. *Neuroscience*, *408*, 1-13. https://doi.org/10.1016/j.neuroscience.2019.03.059

Suslow, T., Kugel, H., Rauch, A. V., Dannlowski, U., Bauer, J., Konrad, C., ... & Ohrmann, P. (2009). Attachment avoidance modulates neural response to masked facial emotion. *Human brain mapping*, *30*(11), 3553-3562. <https://doi.org/10.1002/hbm.20778>

Tseng, W. L., Thomas, L. A., Harkins, E., Pine, D. S., Leibenluft, E., & Brotman, M. A. (2016). Neural correlates of masked and unmasked face emotion processing in youth with severe mood dysregulation. *Social cognitive and affective neuroscience*, *11*(1), 78-88. <https://doi.org/10.1093/scan/nsv087>

Vuilleumier, P., Armony, J. L., Driver, J., & Dolan, R. J. (2001). Effects of attention and emotion on face processing in the human brain: an event-related fMRI study. *Neuron*, *30*(3), 829-841. <https://doi.org/10.1016/S0896-6273(01)00328-2>

Williams, L. M., Liddell, B. J., Kemp, A. H., Bryant, R. A., Meares, R. A., Peduto, A. S., & Gordon, E. (2006). Amygdala–prefrontal dissociation of subliminal and supraliminal fear. *Human brain mapping*, *27*(8), 652-661. https://doi.org/10.1002/hbm.20208

Yang, J., Cao, Z., Xu, X., & Chen, G. (2012). The amygdala is involved in affective priming effect for fearful faces. *Brain and cognition*, *80*(1), 15-22. <https://doi.org/10.1016/j.bandc.2012.04.005>
